# Trajectory of confirmed cases and deaths: fourth wave of COVID-19 epidemic in Myanmar

**DOI:** 10.1186/s12985-023-01960-0

**Published:** 2023-01-07

**Authors:** Ye Minn Htun, Thinzar Aung, Myo Su Kyi, Nyan Htet Shan, Zin Thu Winn, Kaung Si Thu, Nyan Lin Maung, Tun Tun Win, Kyaw Myo Tun

**Affiliations:** 1 Department of Prevention and Research Development of Hepatitis, AIDS and Other Viral Diseases, Health and Disease Control Unit, Nay Pyi Taw, 15011 Myanmar; 2 Department of Public Health, Ministry of Health, Nay Pyi Taw, Myanmar; 3 Outpatient Department, No. 1 Military Hospital (500 Bedded), Meiktila, Mandalay, Myanmar; 4 Department of Research and Development, Defence Services Medical School, Hmawbi, Yangon, Myanmar; 5 Department of Preventive and Social Medicine, Defence Services Medical Academy, Mingaladon, Yangon, Myanmar

**Keywords:** COVID-19, Fourth wave, Mitigation measures, Public health, Vaccination

## Abstract

The current coronavirus disease 2019 (COVID-19) pandemic caused by severe acute respiratory syndrome coronavirus 2 has affected day-to-day life worldwide and presents an unprecedented challenge to public health. Many countries performed mitigation measures to contain the disease spread and break the exponential curve. Omicron had already become a dominant variant in Myanmar and then, the fourth wave of the COVID-19 epidemic started on 28th January 2022. Myanmar performed the main community mitigation measures such as strict quarantine for the people who came back from foreign countries, expansion of testing capacity, enforcement of non-pharmaceutical interventions, and improvement of COVID-19 vaccination coverage. Although decreasing the number of COVID-19 cases and deaths, Myanmar is facing the challenges such as human resource shortages in the health sector, community trust for vaccine safety, and inequitable vaccine demand. This communication intends to give insights on what should be considered as the proper mitigation measures to contain the disease spread through the community and as the challenges that occur in implementing public health and social measures.

## Background

**Fig. 1:**
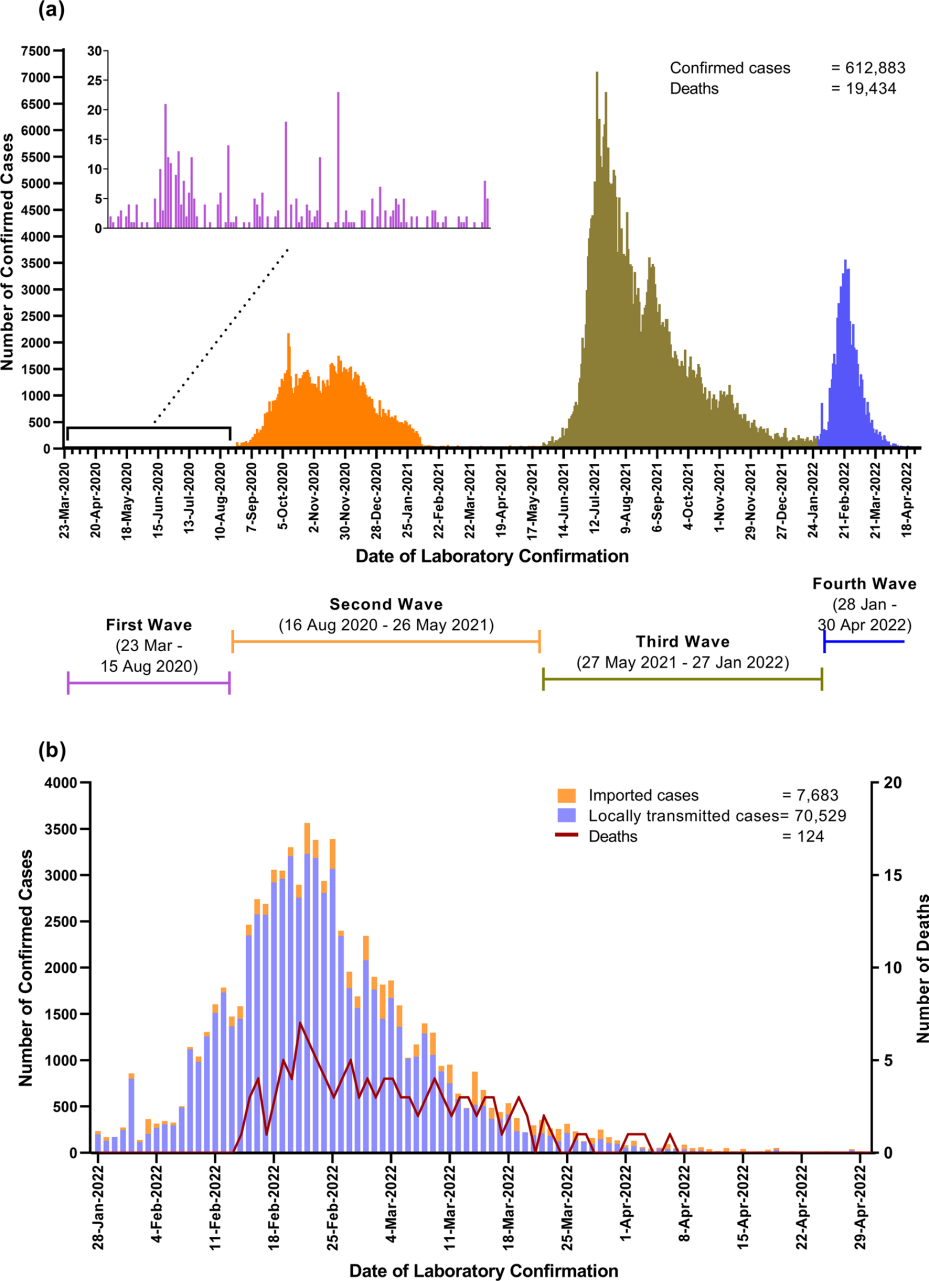
Epidemic curves of COVID-19 in Myanmar. **a** Different waves of COVID-19 epidemic as of 30th April 2022. **b** Fourth wave of COVID-19 epidemic with 7,683 imported case, 70,529 locally transmitted cases, and 124 deaths, from 28th January to 30th April 2022

In the current world, many countries are experiencing different waves of coronavirus disease 2019 (COVID-19) due to the respective dominant variants of severe acute respiratory syndrome coronavirus 2 (SARS-CoV-2) [1]. In Myanmar, there were 612,883 confirmed cases of COVID-19, including 19,434 deaths as of 30th April 2022. The first cases of COVID-19 were reported on 23rd March 2020 and the epidemic started its exponential growth in April 2020 [2, 3]. Incidence of COVID-19 cases and deaths per 100,000 population were expressed in accordance with the total population of the 2014 National Census in Myanmar (51,486,253 persons) [4]. Ministry of Health (MOH) reported that there were 374 confirmed cases (0.73 per 100,000 population) and 6 deaths (0.01 per 100,000 population) with 1.60% case fatality rate (CFR) during the first wave of the epidemic. The second wave started in mid-August 2020 in Rakhine State and the disease spread to the whole country. There were 142,944 confirmed cases (278 per 100,000 population) with 3,210 deaths (6 per 100,000 population) and 2.25% CFR during the second wave [3]. Myanmar has also faced a rapid-surged third wave which started at the end of May 2021. Delta variant rapidly spread throughout the country with the highest impact on lives and the economy. In the third wave, there were 391,353 confirmed cases (760 per 100,000 population) and 16,094 deaths (31 per 100,000 population) with 4.11% CFR (Fig. 1a).

Myanmar launched the COVID-19 vaccination program in January 2021 according to the National Deployment Plan (NDP) [2, 5]. COVID-19 vaccines currently used in Myanmar are COVISHIELD™, BIBP (Sinopharm), Sinovac, COVAXIN, Sputnik Light, and AstraZeneca. At the end of December 2021, 15.6 million people have been fully vaccinated and 5.2 million people were administered first doses of the COVID-19 Vaccine [6]. On 28th December 2021, the Omicron variant (B.1.1.529) was firstly detected in 4 confirmed cases who returned from Dubai, the United Arab Emirates. After detection of Omicron variants, the confirmed cases surged again starting from 28th January 2022, particularly in Yangon Region, and then the fourth wave of COVID-19 was started in Myanmar. The highest number of confirmed cases (3,563) and deaths (7) were reported in the fourth week of February 2022 (Fig. 1b).

## Main text

MOH has been reporting to the public about the daily confirmed cases [tested by reverse transcription polymerase chain reaction (RT-PCR) and rapid diagnostic test] and deaths, risk communication messages, updated information including COVID-19 vaccination, updated standard operating procedures and guidelines regarding prevention and control measures, and health education facts on the official web page (www.mohs.gov.mm) and social media page (www.facebook.com/MinistryOfHealthMyanmar). People who enter from foreign countries through the international airports and ground crossings have to follow the strict quarantine and COVID-19 testing procedures. MOH is also implementing the mitigation measures such as the expansion of testing capacity in both public and private sectors, development of the teleconsultation teams with 24-hour service for people isolated at home, the establishment of rapid response teams linking with COVID-19 test centers and hospital bed management committee, and providing of the treatment at designated treatment centers, and vaccination to the people with targeted groups.

As a SARS-CoV-2 strain surveillance, the genomic sequencing was being performed on the RT-PCR positive samples of some local persons and the people who returned from foreign countries by international flights and cross-border points of entry. Samples from the patients who acquired the infection outside of Myanmar were sequenced but no secondary infections. MOH occasionally announced the news of detected COVID-19 variants. On 18th March 2022, BA.2 sublineage of Omicron variant was detected in 31 specimens tested and a total of 433 confirmed cases with Omicron variant were detected up to 30th April 2022. According to the NDP, in January 2022, booster dose vaccination started in high-risk groups such as frontline healthcare workers and people aged 40 years and above. Up to 30th April 2022, 52.7 million of COVID-19 vaccine doses have been administered and there were 22.5 million people who were fully vaccinated, and 6.5 million people administered first doses of COVID-19 vaccine [2, 6]. The confirmed cases and deaths slightly decreased from the fourth week of February 2022. In the fourth wave, there were 78,212 total confirmed cases (152 per 100,000 population) with 124 deaths (0.24 per 100,000 population) and 0.16% CFR as of 30th April 2022. Myanmar’s health sector is still facing some challenges in responding to COVID-19, such as human resource shortage, technical constraints in data management, delay and/or under-reporting from hard-to-reach areas, poor coordination with multiple stakeholders, community trust for vaccine safety, and inequitable vaccine demand between rural and urban and in conflicted areas.

## Conclusion

In case the coronavirus spreads through the population, the new variants will continue to happen, and then other families of the Omicron variant can continue to emerge. Therefore, the continuation of non-pharmaceutical interventions, real-time disease surveillance, rapid case identification and response, implementing health education and health literacy promotion, improving vaccination coverage, and providing booster doses for all prioritized people should perform to reduce the disease spread. Community participation is crucial to the effective prevention and control of COVID-19. This communication can give the benefit that the countries can adapt to the best practices of the COVID-19 mitigation measures from Myanmar to reduce the disease transmission and prepare for the new wave of the pandemic.

## Data Availability

All data used are publicly available, and sources are cited throughout.

## Abbreviations

COVID-19: Coronavirus disease 2019
MOH: Ministry of Health
SARS-CoV-2: Severe Acute Respiratory Syndrome Coronavirus 2
